# Effects of multiple N, P, and K fertilizer combinations on adzuki bean (*Vigna angularis*) yield in a semi-arid region of northeastern China

**DOI:** 10.1038/s41598-019-55997-9

**Published:** 2019-12-19

**Authors:** Zhi-chao Yin, Wen-yun Guo, Jie Liang, Huan-yu Xiao, Xi-yu Hao, An-fu Hou, Xu-xiao Zong, Ting-rui Leng, Ying-jie Wang, Qing-yu Wang, Feng-xiang Yin

**Affiliations:** 1 College of Plant Science, Jilin University, Changchun, Jilin China; 2 Institute of Edible Bean, Baicheng Academy of Agricultural Sciences, Baicheng, Jilin China; 3 Modern Research and Development Center, Agriculture and Agri-food Canada, Modern, Canada; 4 Institute of Crop Sciences, Chinese Academy of Agricultural Sciences, Beijing, China

**Keywords:** Biotechnology, Plant sciences

## Abstract

Nitrogen (N), phosphorus (P), and potassium (K) exert various effects on adzuki bean yields. Our research was conducted in a semi-arid area, and four test sites were established in environments that have chernozem or sandy loam soils. During a five-year period, the effects of N, P, and K fertilizers on yield were comprehensively investigated in field trials (2014–2016) and for model-implementation trials (2017–2018), with models established prior to the latter. In the field trials, 23 treatments comprising different N, P, and K combinations significantly affected both yield and yield components, and regression analysis indicated that the experimental results were suitable for model establishment. The model subsequently demonstrated that the yield and the yield components were more sensitive to N and K fertilizer than to P fertilizer. Moreover, the yield and yield components increased. These yield increases were intense in response to the 0.5 to 1.34 levels in terms of the single effects; interaction effects; and the effects of combinations of N, P, and K fertilizers. Moreover, the effects of combinations of N, P, and K fertilizers were more significant on yield than were the single or interaction effects of N, P, and K fertilizers. The optimal fertilizer combination that resulted in high yields (≥1941.53 kg ha^−1^) comprised 57.23–68.43 kg ha^−1^ N, 36.04–47.32 kg ha^−1^ P_2_O_5_ and 50.29–61.27 kg ha^−1^ K_2_O. The fertilizer combination that resulted in the maximum yield was 62.98 kg ha^−1^ N, 47.04 kg ha^−1^ P_2_O_5_ and 59.95 kg ha^−1^ K_2_O (N:P_2_O_5_:K_2_O = 1:0.75:0.95), which produced the model-expected yield in trials at multiple sites. An economical fertilizer combination was determined on the basis of the best fertilizer measures in consideration of the cost of fertilizer and seed; this combination achieved yields of 2236.17 kg ha^−1^, the profit was 15,653.16 Yuan ha^−1^, and the corresponding rates were 57.60 kg ha^−1^ N, 47.03 kg ha^−1^ P_2_O_5_, and 31.64 kg ha^−1^ K_2_O (N:P_2_O_5_:K_2_O = 1:0.82:0.55).

## Introduction

Adzuki bean (*Vigna angularis*), which is cultivated in more than thirty countries worldwide, is a major leguminous crop species that is widely consumed and used for medicinal purposes^1,2^. Adzuki bean originated in China and is grown mainly in the temperate regions of East Asia, mainly China, Japan and South Korea. This legume is an important export product in China, where the planting area and total production each account for more than one-third of the corresponding global totals^3,4^. In China, the annual average adzuki bean planting area is approximately 250,000 ha. In addition, the total annual harvest in China is approximately 250,000–350,000 tons, and the export volume is approximately 50,000–80,000 tons. China’s total output and export volume of adzuki beans rank first worldwide. However, in China, soil quality, soil fertility and the quantity of arable soil have declined significantly because of an overdependence on fertilizer^5^. Therefore, the balanced uses of nitrogen (N), phosphorus (P), and potassium (K) fertilizers and proper combinations of N, P, and K fertilizers have become necessary.

Crop yields, vegetative growth and reproduction depend on access to adequate supplies of mineral nutrients, and among these nutrients, N, P, and K are essential for high plant productivity^6,7^. N, P, and K applications play important roles in the growth and quantitative characteristics of adzuki bean^8,9^. Adzuki bean has a strong physiological demand for N, and the appropriate application of N fertilizer at different stages can increase nutrient absorption, plant growth, and yields^10–12^. Increasing the amount of N applied during the early growth period promotes vegetative growth, which leads to high yields. As plants grow, the abundance of rhizobia increases, and their ability to fix atmospheric N improves; however, excessive applications of N fertilizer inhibit rhizobial activity and impede flower bud differentiation and yield formation^13,14^. Moreover, P affects root morphology and growth and therefore water and nutrient uptake, resulting in effective drought mitigation and improved yields^15–17^. K plays important roles in plant growth and in nearly all related functions; it contributes greatly to cell osmotic concentrations and the maintenance of stomata guard cell turgor, increases the photosynthesis rate and biomass production, and increases yields^18–21^.

Fertilizer is a necessary input in agricultural production. However, the unscientific use of fertilizers is a serious threat to sustainable agriculture production systems, and excess fertilizer residue disturbs the soil-nutrient balance and inhibits healthy plant growth. Sustainable crop production can be achieved with appropriate fertilizer by changing the amount of fertilizer applied and using suitable fertilizers^22–25^. Appropriate fertilizer refers to the application of fertilizers at the proper rate, time, and place; when fertilizers are applied appropriately to soils and when their application is accompanied by suitable agronomic implementations, crops can respond to NPK fertilizers without other constraints that limit crop growth^26^.

The objective of our research was to explore the effects of reasonable and effective combinations of N, P, and K fertilizers on yields in a semi-arid area via multiple approaches. Our field research involved applications of various combinations of N, P, and K fertilizers and evaluating their effects on yield and yield components. We then constructed a fertilizer–high yield model and used it to investigate correlations among single effects; interaction effects; and the effects of the optimal combinations of N, P, and K fertilizer. Moreover multipoint trials were used to corroborate the results of the optimal fertilizer combination in production.

## Results

### Effects of different combinations of N, P, and K fertilizers on yield and yield components

Differences in the results for each year were not significant; however, the yield in treatments 5 and 14 was significantly (*P* < *0.05*) lower than that in the other treatments, and the yield in treatments 4, 11, 13 and 6–9 was highly significant (*P* < *0.01*) lower than that in the other treatments. With respect to the number of pods per plant, treatments 4–9 and 11–14 yielded significantly (*P* < *0.05*) lower values than did the other treatments, and with respect to the number of seeds per pod, treatments 1, 5, 8, 10 and 15–23 yielded significantly (*P* < *0.05*) lower values than did the other treatments. Additionally, with respect to 100-seed weight, treatments 5–9 and 11–13 yielded significantly (*P* < 0.05) lower values than did the other treatments. The maximum yield was 2311.90 kg ha^−1^ and was attained in response to level 1 of each N, P_2_O_5_ and K_2_O fertilizer component. The maximum number of pods per plant was 28.10 and occurred in response to level 1 of each N, P_2_O_5_ and K_2_O component, and the maximum number of seeds per pod was 9.44 and occurred in response level −1 of N, level 1 of P_2_O_5_ and level −1 of K_2_O. The maximum 100-seed weight was 12.02 g and was attained in response to level 1.68 of N, level 0 of P_2_O_5_ and level 0 of K_2_O (Table 1).

**Table 1:** Primary field data from the quadratic orthogonal rotation combination experimental design.

Fertilizer combinations (kg ha^−1^)				Yield (kg ha^−1^)				Pods per plant (pods)				Seeds per pod (grain)				100-seed weight (g)			
No	X_1_ (N)	X_2_ (P_2_O_5_)	X_3_ (K_2_O)	2014	2015	2016	Average	2014	2015	2016	Average	2014	2015	2016	Average	2014	2015	2016	Average
1	1 (62.98)	1 (47.04)	1 (59.95)	2301.80	2305.50	2328.40	2311.90 aA	28.56	27.21	28.53	28.10 aA	8.56	7.96	7.60	8.04cdeBC	11.72	11.59	11.79	11.7abAB
2	1 (62.98)	1 (47.04)	−1 (15.25)	2096.60	2081.10	2066.80	2081.50efEF	24.67	24.56	24.57	24.63fF	9.22	8.72	9.10	9.01abcABC	11.52	10.78	10.79	11.03deBC
3	1 (62.98)	−1 (11.96)	1 (59.95)	2108.30	2177.70	2209.30	2165.10cdCD	24.89	25.89	24.49	25.10fEF	9.56	8.65	7.90	8.70abcdeABC	11.12	11.64	11.44	11.4bcdAB
4	1 (62.98)	−1 (11.96)	−1 (15.25)	1533.33	1570.60	1566.77	1556.90jkJK	20.78	19.78	20.94	20.50gG	9.78	8.78	9.30	9.29abAB	10.08	10.84	10.70	10.54efCD
5	−1 (16.02)	1 (47.04)	1 (59.95)	1765.60	1880.00	1881.60	1842.40hH	23.11	22.11	22.61	22.60gG	8.00	8.61	8.60	8.40bcdeABC	9.12	9.05	9.85	9.34ijFG
6	−1 (16.02)	1 (47.04)	−1 (15.25)	1696.80	1642.70	1641.70	1660.40iI	20.67	19.67	19.36	19.93hiH	9.11	9.81	9.40	9.44 aA	9.01	9.83	9.66	9.5hijEFG
7	−1 (16.02)	−1 (11.96)	1 (59.95)	1575.00	1550.60	1507.60	1544.40kJK	20.22	20.92	19.97	20.37hH	8.56	8.96	9.10	8.87abcdABC	9.01	9.39	9.05	9.15jG
8	−1 (16.02)	−1 (11.96)	-1 (15.25)	1480.80	1463.50	1483.40	1475.90lmLM	20.33	19.33	19.74	19.77hiH	8.89	8.39	7.82	8.37bcdeABC	9.79	9.23	9.63	9.55hijEFG
9	−1.68 (0)	0 (29.5)	0 (37.6)	1521.70	1507.80	1509.20	1512.90klKL	20.44	19.90	20.08	20.13hiH	9.44	8.94	8.60	8.99abcABC	9.67	9.81	9.80	9.76ghiEFG
10	1.68 (78.99)	0 (29.5)	0 (37.6)	2080.60	2038.80	2059.10	2059.50fgEFG	25.20	24.50	26.11	25.27fEF	8.11	8.71	8.10	8.31bcdeABC	11.94	12.77	11.35	12.02 aA
11	0 (39.5)	−1.68 (0)	0 (37.6)	1605.80	1601.10	1597.60	1601.50iIJ	22.80	22.78	22.82	22.80gG	9.89	8.39	7.80	8.69abcdeABC	9.89	9.70	10.14	9.91ghDEF
12	0 (39.5)	1.68 (59)	0 (37.6)	2167.50	2158.30	2118.50	2148.10cdD	24.44	24.44	24.92	24.57fF	9.44	7.94	8.94	8.77abcdeABC	9.36	9.91	9.53	9.6hijEFG
13	0 (39.5)	0 (29.5)	−1.68 (0)	1454.10	1446.70	1416.50	1439.10 mM	19.56	19.16	19.12	19.30iH	9.56	8.86	9.87	9.43 aA	9.96	10.03	10.52	10.17fgDE
14	0 (39.5)	0 (29.5)	1.68 (75.19)	1898.33	1809.40	1810.47	1839.40hH	22.67	21.67	23.22	22.53gG	9.67	7.67	8.30	8.55abcdeABC	11.25	10.97	10.96	11.06cdeBC
15	0 (39.5)	0 (29.5)	0 (37.6)	2066.60	2028.30	2040.83	2045.23fgFG	26.20	26.80	26.50	26.50cdBCD	7.78	8.58	7.00	7.79eC	11.19	11.08	11.87	11.38bcdAB
16	0 (39.5)	0 (29.5)	0 (37.6)	2286.50	2293.80	2257.50	2279.27aAB	27.60	27.20	26.80	27.20abcABC	7.44	7.94	8.00	7.79eC	11.02	11.32	11.98	11.44bcdAB
17	0 (39.5)	0 (29.5)	0 (37.6)	2170.80	2080.30	2104.17	2118.43deDE	25.56	25.56	25.08	25.43efDEF	7.33	8.83	7.90	8.02cdeBC	11.69	11.99	11.57	11.75abAB
18	0 (39.5)	0 (29.5)	0 (37.6)	2258.30	2202.20	2211.67	2224.07bBC	26.80	25.10	26.70	26.20deCDE	7.11	8.61	8.10	7.94deC	11.57	11.74	11.70	11.67abAB
19	0 (39.5)	0 (29.5)	0 (37.6)	2180.80	2181.60	2176.67	2179.70bcCD	27.01	28.67	27.42	27.70abAB	7.67	8.67	7.60	7.98deBC	11.24	10.86	10.96	11.02deBC
20	0 (39.5)	0 (29.5)	0 (37.6)	2150.60	2187.50	2160.83	2166.30cCD	26.33	26.33	26.24	26.27deCDE	7.33	8.33	8.20	7.95deBC	11.24	11.50	10.98	11.24bcdBC
21	0 (39.5)	0 (29.5)	0 (37.6)	2238.30	2216.10	2208.33	2220.90bBC	27.12	27.78	26.40	27.10bcdABC	7.44	8.74	8.40	8.19cdeABC	10.84	11.49	11.87	11.4bcdAB
22	0 (39.5)	0 (29.5)	0 (37.6)	2187.50	2145.50	2161.67	2164.90cdCD	26.30	26.33	26.27	26.30cdeCDE	7.78	8.78	8.60	8.39bcdeABC	11.54	11.47	11.82	11.61abAB
23	0 (39.5)	0 (29.5)	0 (37.6)	2005.90	2010.80	2035.70	2017.47gG	27.23	26.89	25.98	26.70cdBC	8.00	8.32	8.00	8.11cdeABC	11.49	10.92	11.28	11.23bcdBC

The differences among years are not significant, with *P*-values of 0.3082, 0.2655, 0.4782 and 0.2183; the differences in each treatment are significant, with *P*-values of 0.0001, 0.0001, 0.0168 and 0.0001. The differences in the mean values of each treatment indicated by a, b and c are significant (*P* < 0.05). The differences in the mean values of each treatment indicated by A, B and C are highly significant (*P* < 0.01).

### Regression-based relationships between N, P, and K fertilizers and yield and yield components

Regression analysis based on the field research yielded the following four equations for expected yield (1), number of pods per plant (2), number of seeds per pod (3), and 100-seed weight (4) in response to N, P, and K fertilizer.

1 $$\begin{array}{rcl}{\rm{Y}} & = & 2155.50+183.91{{\rm{X}}}_{1}+151.80{{\rm{X}}}_{2}+129.04{{\rm{X}}}_{3}-113.24{{\rm{X}}}_{1}^{2}-81.91{{\rm{X}}}_{2}^{2}\\  &  & -\,165.19{{\rm{X}}}_{3}^{2}+23.61{{\rm{X}}}_{1}{{\rm{X}}}_{2}+73.51{{\rm{X}}}_{1}{X}_{3}-33.04{{\rm{X}}}_{2}{{\rm{X}}}_{3}\end{array}.$$

2 $$\begin{array}{rcl}{\rm{Y}} & = & 26.59+1.78{{\rm{X}}}_{1}+0.91{{\rm{X}}}_{2}+1.23{{\rm{X}}}_{3}-1.29{{\rm{X}}}_{1}^{2}-0.93{{\rm{X}}}_{2}^{2}\\  &  & -\,1.92{{\rm{X}}}_{3}^{2}+0.60{{\rm{X}}}_{1}{{\rm{X}}}_{2}+0.60{{\rm{X}}}_{1}{{\rm{X}}}_{3}+0.13{{\rm{X}}}_{2}{{\rm{X}}}_{3}.\end{array}$$

3 $$\begin{array}{rcl}{\rm{Y}} & = & 8.02-0.09{{\rm{X}}}_{1}-0.02{{\rm{X}}}_{2}-0.26{{\rm{X}}}_{3}+0.21{{\rm{X}}}_{2}^{2}+0.24{{\rm{X}}}_{2}^{2}\\  &  & +\,0.33{{\rm{X}}}_{3}^{2}-0.19{{\rm{X}}}_{1}{{\rm{X}}}_{2}-0.13{{\rm{X}}}_{1}{{\rm{X}}}_{3}-0.24{{\rm{X}}}_{2}{{\rm{X}}}_{3}.\end{array}$$

4 $$\begin{array}{rcl}{\rm{Y}} & = & 11.42+0.80{{\rm{X}}}_{1}+0.03{{\rm{X}}}_{2}+0.18{{\rm{X}}}_{3}-0.20{{\rm{X}}}_{1}^{2}-0.60{{\rm{X}}}_{2}^{2}\\  &  & -\,0.30{{\rm{X}}}_{3}^{2}+0.08{{\rm{X}}}_{1}{{\rm{X}}}_{2}+0.26{{\rm{X}}}_{1}{{\rm{X}}}_{3}+0.01{{\rm{X}}}_{2}{{\rm{X}}}_{3}.\end{array}$$

The regression analysis results are shown in Table 2. All the *P*-values of the above four equations were 0.0001, indicating significance (*P* < 0.01); thus, the mathematical models exhibit good adaptability. The *P*-values of the misfit for each equation were 0.2073 (1), 0.1437 (2), 0.1833 (3), and 0.2499 (4) and were not significant (*P* > 0.05); these results indicated that the influences of external factors on the experimental results could be ignored. Therefore, the four regression models were suitable for assessing the effects of N, P and K fertilizers on yield and yield components.

**Table 2:** Analysis of variance of the effects of N, P, and K fertilizers on yield and yield components.

Source of variation	Degrees of freedom	Yield			Number of pods per plant			Number of seeds per pod			100- seed weight		
		Mean square	F-value	*P*-value	Mean square	F-value	*P*-value	Mean square	F-value	*P*-value	Mean square	F-value	*P*-value
X_1_	1	461890.67	50.7220	0.0001**	43.40	43.3991	0.0001**	0.1026	0.1026	0.1665	8.7490	133.0248	0.0001**
X_2_	1	314715.62	34.5601	0.0001**	11.31	11.3083	0.001**	0.0031	0.0031	0.8031	0.0122	0.1859	0.6734
X_3_	1	227415.30	24.9734	0.0002**	20.62	20.6216	0.0001**	0.9384	0.9384	0.0007**	0.4456	6.7747	0.0219*
X_1_^2^	1	197704.62	21.7107	0.0005**	26.31	26.3067	0.0001**	0.6916	0.6916	0.0022**	0.6554	9.9653	0.0076*
X_2_^2^	1	101704.29	11.1685	0.0053**	13.84	13.8361	0.0004**	0.8918	0.8918	0.0008**	5.8040	88.2475	0.0001**
X_3_^2^	1	426632.15	46.8501	0.0001**	58.76	58.764	0.0001**	1.7182	1.7182	0.0001**	1.4331	21.7903	0.0004*
X_1_X_2_	1	4460.40	0.4898	0.4963	2.88	2.88	0.0519*	0.2964	0.2964	0.027*	0.0528	0.8030	0.3865
X_1_X_3_	1	43232.70	4.7475	0.0483*	2.88	2.88	0.0519*	0.1300	0.1300	0.1228	0.5460	8.3019	0.0129*
X_2_X_3_	1	8731.81	0.9589	0.3453	0.13	0.125	0.6630	0.4608	0.4608	0.0083**	0.0003	0.0048	0.9461
Regression	9	1795414.52	F2 = 21.907	0.0001**	178.94	F_2_ = 31.6273	0.0001**	5.1906	F_2_ = 12.0804	0.0004**	17.6195	F_2_ = 29.7664	0.0001**
Residual	13	118382.14			8.17			0.6206			0.8550		
Lack of fit	5	60726.80	F1 = 1.685	0.2073	4.55	F_1_ = 2.0120	0.1437	0.3281	F_1_ = 1.7943	0.1833	0.4168	F_1_ = 1.5217	0.2499
Error	8	57655.34			3.62			0.2926			0.4382		
Total	22	1913796.83			187.11			5.8112			18.4745		

The data shown are from the results calculated by software. X_1,_ X_2,_ and X_3_ represent N, P, and K fertilizers, respectively. *Indicates significance (*P* < 0.05), and **indicates high significance (*P* < 0.01).

According to the absolute values of the regression coefficients of the above four equations, the relative magnitudes of the effects of N, P, and K fertilizers on yield, number of pods per plant, number of seeds per pod, and 100-seed weight were N > P > K (1), N > K > P (2), K > N > P (3), and N > K > P (4), respectively. The relative effect sizes of the interactions of N, P and K fertilizers on yield, number of pods per plant, number of seeds per pod and 100-seed weight were NK > NP > PK (1), NP >  = NK > PK (2), PK > NP > NK (3), and NK > NP > PK (4), respectively.

### Single effects of N, P, and K fertilizers on yield and yield components

**Figure 1:**
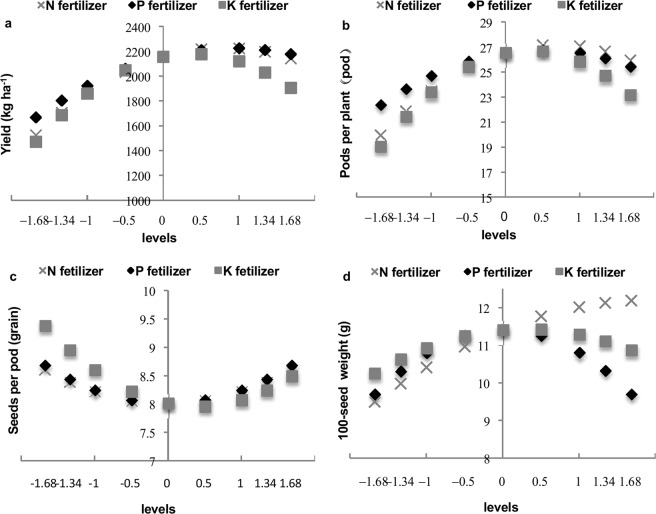
Single effect of N, P, and K fertilizers on yield and yield components. The vertical axes represent the yield, number of pods per plant, number of seeds per pod and 100-seed weight; the lateral axis interval represents fertilizer levels; and the series represents the N, P, and K fertilizers. (**a**) Single effects of N, P, and K fertilizers on yield. (**b**) Single effect of N, P, and K fertilizers on the number of pods per plant (**c**) Single effects of N, P, and K fertilizers on the number of seeds per pod. (**d**) Single effect of N, P, and K fertilizers on 100-seed weight.

N, P and K fertilizers had highly significant effects (*P* < 0.01) on both yield and number of pods per plant (Table 2). As shown in Fig. 1a,b, however, the yield and number of pods per plant increased to a significantly greater degree in response to N and K fertilizers at levels of −1.68 to 0.5, and the effect of increased P fertilizer on the two indexes was relatively smooth. However, at fertilizer levels of 0.5 to 1.68, unlike the pattern observed in response to N and P fertilizers, the yield and number of pods per plant strongly decreased with increasing K fertilizer. The increased yield and number of pods per plant were attained in response to N and P levels of −0.5 to 1.68 and K fertilizer levels of −0.5 to 1.34.

K fertilizer had a significant (*P* < 0.01) effect on number of seeds per pod, whereas the N and P fertilizers had no significant (*P* > 0.05) effect on this variable (Table 2). As shown in Fig. 1c, the effects of the N, P, and K fertilizers on the number of seeds per pod were not significant; the number of seeds per pod remained at similar values between the fertilizer levels of −1.68 to 0 and 0 to 1.68.

N and K fertilizers had significant (*P* < 0.01) effects on 100-seed weight, whereas P fertilizer did not (*P* > 0.05) (Table 2). As shown in Fig. 1d, N, P, and K fertilizers at levels of −1.68 to −1 had inconspicuous effects on 100-seed weight; however, at N, P, and K levels of −0.5 to 1.68, the effects on 100-seed weight were much greater, especially in response to the N and K fertilizers.

### Interaction effects of N, P, and K fertilizers on yield and yield components

**Figure 2:**
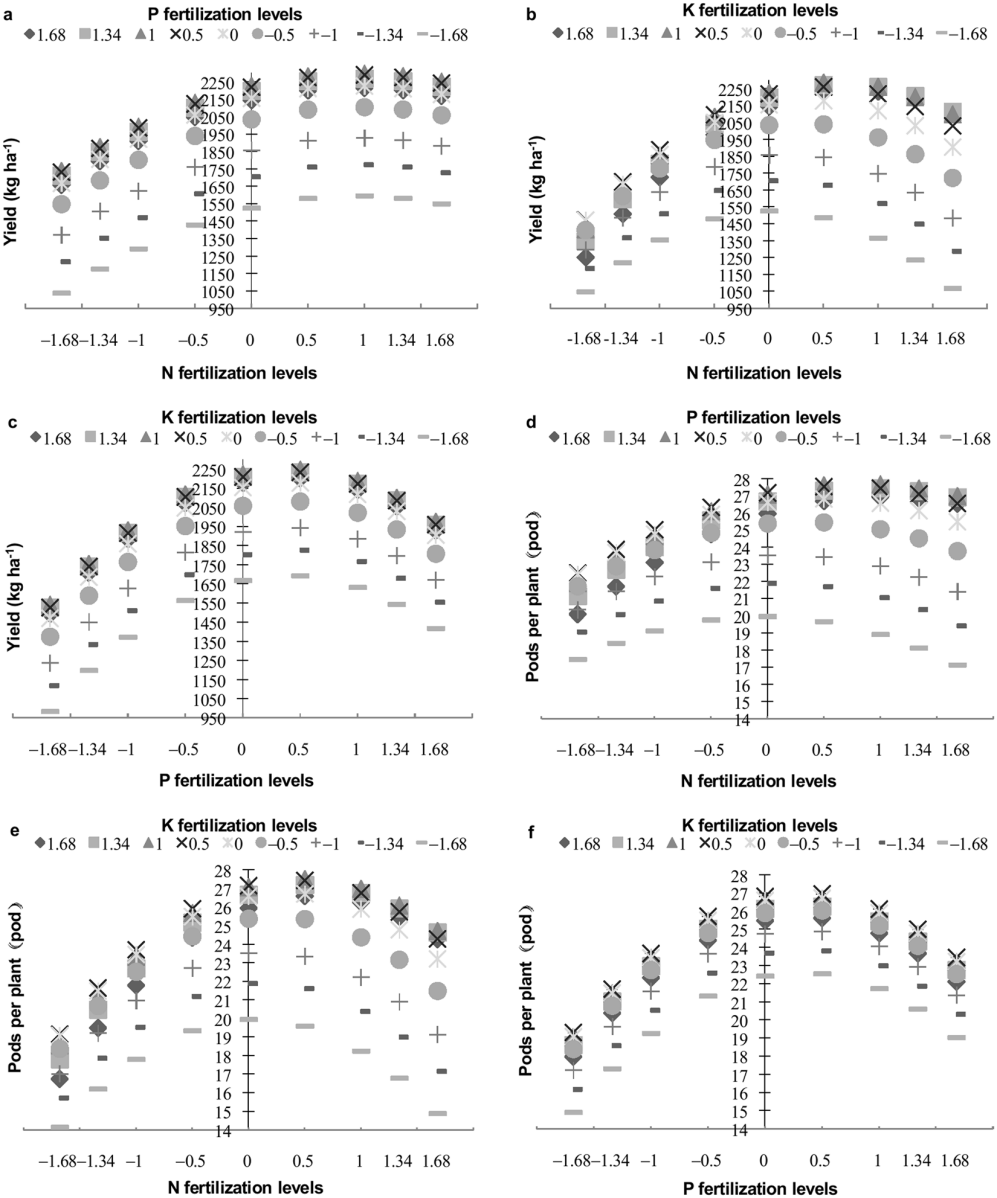
Interaction effects of N, P, and K on yield and the number of pods per plant. The vertical line represents the values of yield and pods per plant. (**a**) Interaction effects of N and P on yield. (**b**) Interaction effects of N and K on yield. (**c**) Interaction effects of P and K on yield. (**d**) Interaction effects of N and P on the number of pods per plant. (**e**) Interaction effects of N and K on the number of pods per plant. (**f**) Interaction effects of P and K on the number of pods per plant.

**Figure 3:**
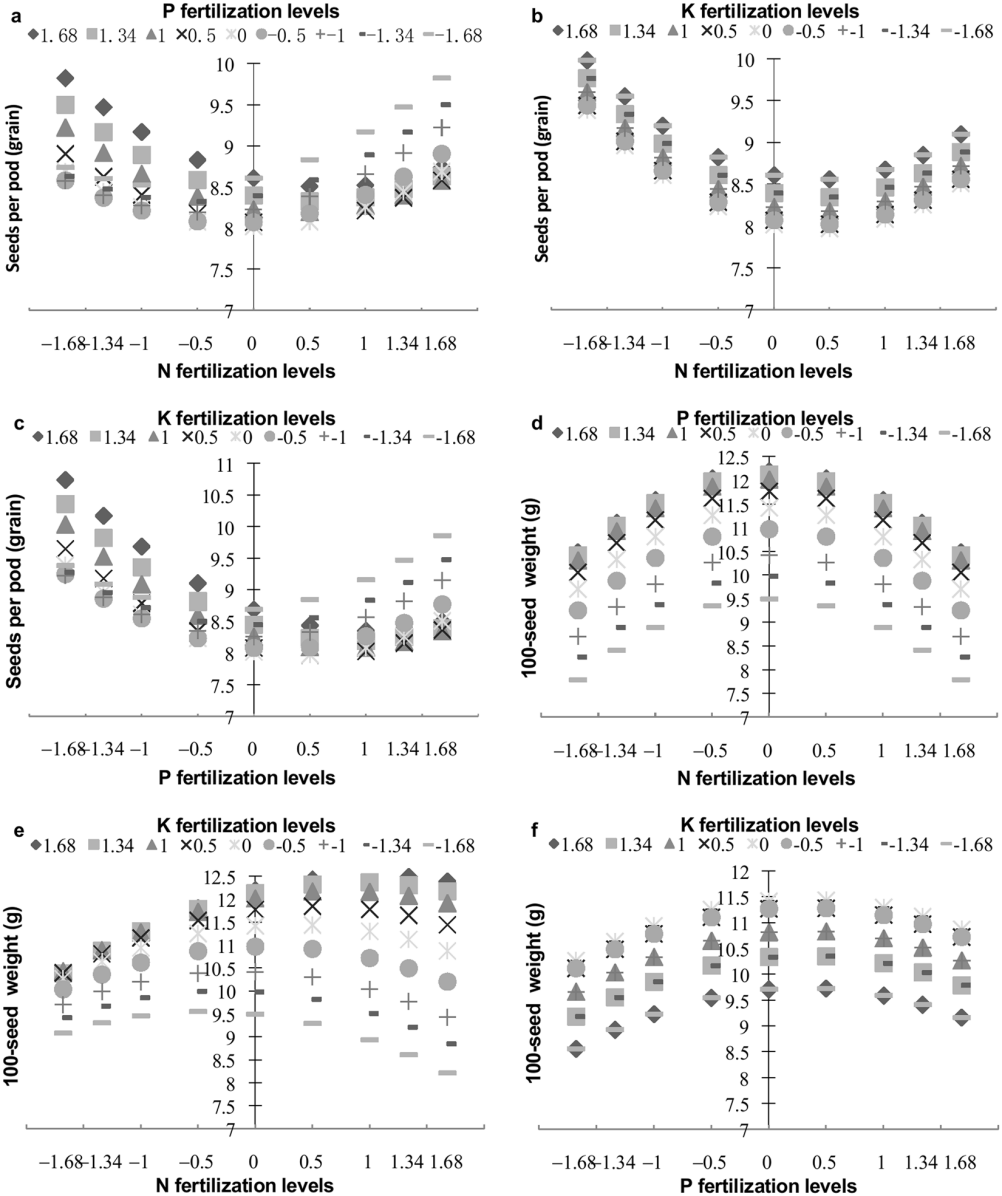
Interaction effects of N, P, and K on the number of seeds per pod and 100-seed weight. The vertical line represents the values of seeds per pod and 100-seed weight (**a**) Interaction effects of N and P on seeds per pod. (**b**) Interaction effects of N and K on seeds per pod. (**c**) Interaction effects of P and K on seeds per pod. (**d**) Interaction effects of N and P on 100-seed weight. (**e**) Interaction effects of N and K on 100-seed weight. (**f**) Interaction effects of P and K on 100-seed weight.

The interaction between N and K fertilizers had significant (*P* < 0.05) effects on the yield, number of pods per plant and 100-seed weight. However, the interactions between P and K fertilizers had no significant (*P* > 0.05) effects on these variables, and the interactions between N and P fertilizers had no significant effect on yield or 100-seed weight (Table 2). The N and K interaction had a significant effect on the yield, number of pods per plant and 100-seed weight in response to N and K fertilizer levels ranging from −0.5 to 1.68 (Figs. 2b,e and 3e).

The interaction between N and P had significant (*P* < 0.05) effects on the number of pods per plant and the number of seeds per pod. Moreover, the interaction between P and K had highly significant (*P* < 0.01) effects on the number of seeds per pod, whereas that between N and K did not (*P* > 0.05) (Table 2). The interaction between N and P had a prominent effect on the number of pods per plant at N and P fertilizer levels ranging from −0.5 to 1.68 (Fig. 2d). With respect to the number of seeds per pod, N at levels ranging from −1.68 to 0 interacted strongly with P at levels ranging from 0 to 1.68, and N at levels from 0 to 1.68 interacted strongly with P at levels from −1.68 to 0. Similarly, P at levels ranging from −1.68 to 0 interacted strongly with K at levels from 0 to 1.68, and P at levels of 0 to 1.68 interacted obviously with K at levels of −1.68 to 0. However, the interaction effects of N and P and of P and K on the number of seeds per pod exerted no pronounced differences at high or low levels of N and P fertilizers and of P and K fertilizers, respectively (Fig. 3a,c).

### Optimal fertilizer combinations

According to the 95% confidence intervals for yield, the N fertilizer interval for high yield (≥1941.53 kg ha^−1^) ranged from 57.23–68.43 kg ha^−1^, and the corresponding N fertilizer levels was 0.75 to 1.23, which lay within the range of the 0.5 to 1.34 levels. The P fertilizer interval was 36.04–47.32 kg ha^−1^, which corresponded to P fertilizer levels of 0.37 to 1.01 and was located within the range of the 0 to 1 levels, and the K fertilizer interval was 50.29–61.27 kg ha^−1^ and corresponded to K levels of 0.56 to 1.06, corresponding to the 0.5 to 1.34 levels (Table 3). The 95% confidence intervals for number of pods per plant revealed that the N fertilizer interval for a relatively high numbers of pods per plant (≥24.13) was 56.55–67.91 kg ha^−1^, corresponding to 0.73 to 1.20, which was located within the 0.5 to 1.34 levels. Similarly, the P fertilizer interval was 35.66–47.32 kg ha^−1^, corresponding to 0.35 to 1.01, which lied within the 0 to 1 fertilizer level range. Last, the K fertilizer interval was 48.60–59.81 kg ha^−1^, and the corresponding levels were 0.49 to 0.99, representing the fertilizer levels of 0.5 to 1 (Table 3).

**Table 3:** Frequency of factors composing fertilizer combinations for high yields and yield components.

Yield and yield components	Items	N	P_2_O_5_	K_2_O
High yield (≥1941.53 kg ha^−1^)	Ymax = 2333.42 kg ha^−1^	X_1_ = 1	X_2_ = 1	X_3_ = 1
	Weight mean	0.9930	0.6950	0.8310
	Standard error	0.1220	0.1640	0.1250
	95%confidenceinterval	0.755–1.232	0.373–1.016	0.568–1.059
	Higher yield fertilization	57.23–68.43	36.04–47.32	50.29–61.27
High number of Pods per plant (≥24.13)	Ymax = 27.66	X_1_ = 1	X_2_ = 1	X_3_ = 0
	Weight mean	0.968	0.683	0.744
	Standard error	0.124	0.17	0.128
	95%confidenceinterval	0.726–1.210	0.351–1.016	0.492–0.996
	Fertilization(kg ha^−1^)	56.55–67.91	35.66–47.32	48.60–59.81
High number of Seeds per pod (≥8.48)	Ymax = 11.87	X_1_ = −1.68	X_2_ = −1.68	X_3_ = −1
	Weight mean	−0.069	−0.111	−0.133
	Standard error	0.125	0.123	0.124
	95%confidenceinterval	−0.314–0.175	−0.352–0.130	−0.375–0.110
	Fertilization(kg ha^−1^)	32.13–43.61	23.33–31.78	29.22–40.06
High100-seed weight (≥10.76 g)	Ymax = 12.51	X_1_ = 1.68	X_2_ = 0	X_3_ = 1
	Weight mean	1.078	1	0.635
	Standard error	0.121	0.158	0.162
	95%confidenceinterval	0.841–1.314	−0.310–0.310	−0.318–0.952
	Fertilization(kg ha^−1^)	59.24–70.35	24.06–34.94	30.49–58.88

The frequency table was from the software analysis.

The 95% confidence intervals for the 100-seed weight indicated that, to achieve a high 100-seed weight (≥10.76), an N fertilizer amount of 59.24–70.35 kg ha^−1^ was needed, which was within the levels of 0.49 to 1.31, likely corresponding to the levels of 0.5 to 1.34. The P fertilizer amount needed (24.06–34.94 kg ha^−1^) was achieved in response to levels of −0.31 to 0.31, corresponding to the −0.5 to 0.5 interval, and the K fertilizer amount needed (30.49–58.88 kg ha^−1^) was achieved in response to levels of −0.31 to 0.95, corresponding to levels of −0.5 to 1 (Table 3).

The 95% confidence intervals indicated that the N, P, and K fertilizer intervals for a high number of seeds per pod (≥8.48) were 32.13–43.61 kg ha^−1^, 23.33–31.78 kg ha^−1^, and 29.22–40.06 kg ha^−1^, respectively, corresponding to levels of −0.37 to 0.17, −0.35 to 0.12, and −0.37 to 0.11, respectively, which were within the levels of −0.5 to 0.5 (Table 3).

### Fertilizer combinations for maximum yield and yield components

**Figure 4:**
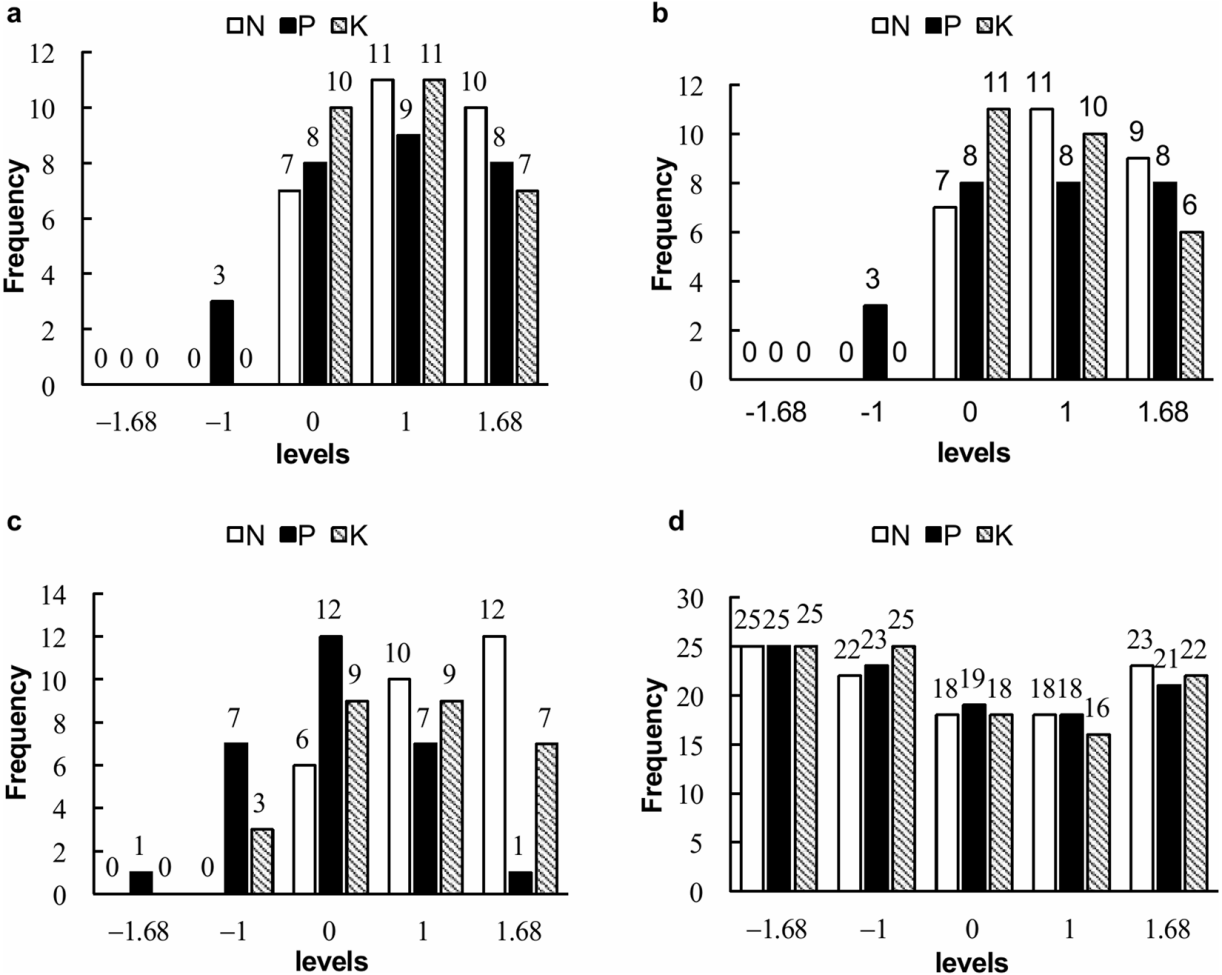
Frequency distribution of N, P, and K in the combinations resulting in high values. (**a**) Frequency distribution of N, P, and K in 28 combinations resulting in yields greater than 1941.53 kg ha^−1^. (**b**) Frequency distribution of N, P, and K in 27 combinations resulting in more than 24.13 pods per plant. (**c**) Frequency distribution of N, P, and K in 106 combinations resulting in more than 8.48 seeds per pod. (**d**) Frequency distribution of N, P, and K in 28 combinations a 100-seed weight greater than 10.76 g.

Frequency analysis revealed 28 fertilizer combinations that resulted in high yields ( > 1941.53 kg ha^−1^) and 27 combinations for achieving high number of pods per plant ( > 24.13). Under these combinations, the effects of N, P, and K on the yield and number of pods per plant were apparent primarily at the 0 to 1.68 levels. The most influential fertilizer levels included level 1 for N (62.98 kg ha^−1^), P_2_O_5_ (47.04 kg ha^−1^), and K_2_O (59.95 kg ha^−1^) for yield and included level 1 for N (62.98 kg ha^−1^) and P_2_O_5_ (47.04 kg ha^−1^) but level 0 for K_2_O (37.60 kg ha^−1^) for number of pods per plant (Fig. 4). The combinations with the most influential components among the three fertilizers generated the greatest yield (2333.42 kg ha^−1^) and number of pods per plant (27.66) (Table 3).

With respect to 100-seed weight, there were 28 fertilizer combinations that resulted in high values ( > 10.76); for these combinations, the firm range for N and K fertilizers was 0 to 1.68 and that for P fertilizer was −1 to 1. The most influential fertilizer comprised N at the 1.68 level (78.99 kg ha^−1^), a P_2_O_5_ at the 0 level (29.50 kg ha^−1^), and K_2_O at the 1 level (59.95 kg ha^−1^) (Fig. 4); the greatest 100-seed weight (12.51 g) was achieved with the combination of the same three fertilizer levels (Table 3).

However, with respect to the number of seeds per pod, there were 106 fertilizer combinations that achieved high values (≥8.48), and the results under the high fertilizer conditions and the no N, P, or K fertilizer conditions were similar (Fig. 4). The maximum number of seeds per pod was 11.87 at the −1.68 level (0 kg ha^−1^) of N, the −1.68 level (0 kg ha^−1^) of K_2_O and the −1 level (15.25 kg ha^−1^) of K_2_O (Table 3).

### Economical fertilizer programme

To identify the most economical fertilizer programme, the cost of fertilizer and the price of adzuki beans must be considered. The price of adzuki beans in this study was 7 Yuan kg^−1^. After the cost of fertilizer was removed, the equation for the net profit substitution model was calculated as follows:

5 $$\begin{array}{rcl}{\rm{Y}} & = & (2155.50+183.91{{\rm{X}}}_{1}+151.80{{\rm{X}}}_{2}+129.04{{\rm{X}}}_{3}-113.24{{\rm{X}}}_{1}^{2}\\  &  & -\,81.91{{\rm{X}}}_{2}^{2}-165.19{{\rm{X}}}_{3}^{2}+23.61{{\rm{X}}}_{1}{{\rm{X}}}_{2}+73.51{{\rm{X}}}_{1}{{\rm{X}}}_{3}-33.04{{\rm{X}}}_{2}{{\rm{X}}}_{3})\\  &  & \times \,7-(156.82+93.22{{\rm{X}}}_{1})-(117.59+105.59{{\rm{X}}}_{2})-(203.79+121.14{{\rm{X}}}_{3}).\end{array}$$

The simplified equation is as follows:

6 $$\begin{array}{rcl}{\rm{Y}} & = & 14,610.3+1194.15{\rm{X}}1+957.01{{\rm{X}}}_{2}-782.14{{\rm{X}}}_{3}-792.7{{\rm{X}}}_{1}^{2}-573.37{{\rm{X}}}_{2}^{2}\\  &  & -\,1156.33{{\rm{X}}}_{3}^{2}+165.27{{\rm{X}}}_{1}{{\rm{X}}}_{2}+514.57{{\rm{X}}}_{1}{{\rm{X}}}_{3}-231.3{{\rm{X}}}_{2}{{\rm{X}}}_{3}.\end{array}$$

Partial equations of the above equations were obtained. The partial derivatives of the elements X_1_ (N), X_2_ (P), and X_3_ (K) were equal to zero, and the following equations were obtained:

$$\{\begin{array}{rcl}1194.15-1585.4{{\rm{X}}}_{1}+165.27{{\rm{X}}}_{2}+514.57{X}_{3} & = & 0\\ 957.01-1146.74{{\rm{X}}}_{2}+165.27{{\rm{X}}}_{1}-231.30{{\rm{X}}}_{3} & = & 0\\ -782.14-2312.66{{\rm{X}}}_{3}+514.57{{\rm{X}}}_{1}-231.30{{\rm{X}}}_{2} & = & 0\end{array}$$

Under the conditions −1.68 ≤ xi ≤ 1.68, the most economically beneficial bean fertilizer combination was determined. By determining that X_1_ = 0.7709, X_2_ = 0.9994, and X_3_ = −0.2666 and inserting these values into the formula, we obtained values of 57.60 kg/ha^−1^ N, 47.03 kg/ha^−1^ P_2_O_5_ and 31.64 kg/ha^−1^ K_2_O for the N:P_2_O_5_:K_2_O ratio = 1:0.82:0.55. The optimum fertilizer ratio was therefore 1:0.82:0.55, the resulting yield was 2236.17 kg/ha^−1^, and the profit was 15,653.16 Yuan.

### Comparisons of the optimal fertilizer combination and normal fertilizers

**Figure 5:**
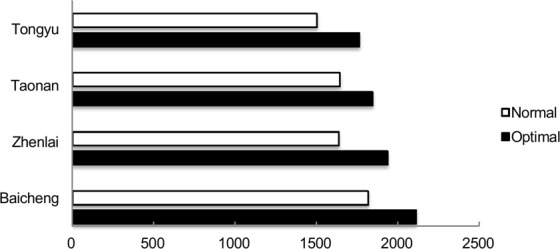
Implementations of the optimal fertilizer model. The trials were conducted at the four locations by comparing the optimal fertilizer combination with normal fertilizer. The data are the average of 2017–2018; the difference between the two years was not significant (*P* < 0.05).

To further test the optimal fertilizer combination obtained in this study, the verification production tests were carried out in Baicheng, Zhenlai, Tongyu and Taonan in Jilin in 2017–2018. Under the relatively high-moisture and high-fertility conditions in Baicheng, compared with normal fertilizer, the optimal fertilizer combination resulted in 16.19% greater yields, which reached 2007.7 kg ha^−1^. Under the lower-fertility conditions of Zhenlai, Taonan and Tongyu, moderate yields were obtained; compared with that in response to normal fertilizer, the average yield (1762.40 kg/ha^−1^) of the three test points was 15.20% greater through optimal fertilizer combinations. Two-year multipoint tests determined an optimal fertilizer programme with an average yield of 1823.76 kg ha^−1^, which was 15.26% higher than that obtained with normal fertilizer (Fig. 5).

## Discussion

Previous researchers on N, P, and K fertilizers applied to leguminous species or other crop species have limited their investigations to short-term data collection with narrow treatment settings for establishing fertilizer combinations, and relatively little data exploration has occurred^26–30^. Hence, research on determining reasonable N, P, and K levels to apply to optimize adzuki bean yield quality is necessary. We explored the effects of N, P, and K fertilizers via multiple approaches. Our primary field research led to the development of a stable database, a fertilizer model was constructed to provide optimal amounts for achieving high yields, and multipoint production trials validated the model predictions.

The quadratic orthogonal rotation combination design applied in the field research was effective for determining the fertilizer combination that resulted in the greatest adzuki bean yield in our study. On the basis of the significant performance of the different combinations of N, P and K fertilizers in the field trail, and the fertilizer combination for achieving maximum yield (2311.90 kg ha^−1^) in the field research was at 1 level of N, P_2_O_5_ and K_2_O. These results constitute a reliable data foundation for model establishment. The fertilizer model was constructed via assessment of the comprehensive performance of single and interaction effects among the various fertilizers. Last, the optimal combination of N, P, and K fertilizer was ultimately obtained.

The single effects of N, P, and K fertilizers in our model indicated that the yield and number of pods per plant were more sensitive to K fertilizer than to N and P fertilizers, and the results indicated that increasing the application of N and K significantly increased the 100-seed weight^31,32^. Moreover, we found that the number of seeds per pod responded significantly only to K, which has also reported for dry bean species^32^. The outstanding performance resulting from K fertilizer revealed that K application might be a viable strategy for improving yields, as reported previously^33^

Previous studies have shown that high numbers of pods per plant, numbers of seeds per pod and 100-seed weight are considered yield quality characteristics of adzuki bean^34^, in our model, these three components increased. Increased yield, number of pods per plant, and 100-seed weight were intensively obtained at the 0.5 to 1.34 levels in response to single and interaction effects of N, P, and K fertilizers. However, with respect to the number of seeds per pod, the effects of N, P, and K fertilizers did not differ between the low and high levels (Figs. 1–3). Frequency analysis of N, P, and K for the optimal fertilizer combination revealed that the values associated with the number of pods per plant and the 100-seed weight were in accordance with those of yield, with influential effects of N, P, and K fertilizers occurring at high levels (0 to 1.68). With respect to the number of seeds per pod, no significant N, P, and K effects were observed between the −1.68 to 0 and 0 to 1.68 intervals. Furthermore, the optimal fertilizer combination of 57.23–68.43 kg ha^−1^ of N, 36.04–47.32 kg ha^−1^ of P_2_O_5_ and 50.29–61.27 kg ha^−1^ of K_2_O for high yield (≥1941.53 kg ha^−1^) corresponded to the 0.5 to 1.34 levels of the N, P, and K fertilizers. Overall, the above results demonstrated that the high yields were achieved under the single and interaction effects and under the effects of optimal combinations of N, P, and K fertilizers at the same interval (0.5 to 1.34 levels). In particular, the combinations of N, P, and K fertilizers had a more significant effect on yield than did the single or interaction effects of N, P, and K fertilizers, which are also indicated in other articles^35,36^.

In our study, the optimal fertilizer combination for maximum yield (2333.42 kg ha^−1^; N:P_2_O_5_:K_2_O = 1:0.75:0.95), 62.98 kg ha^−1^ N, 47.04 kg ha^−1^ P_2_O_5_, and 59.95 kg ha^−1^ K_2_O, was approaching the upper limit of the optimal fertilizer combination. The maximum yields (1225–1750 kg ha^−1^) reported in the literature for subtropical humid regions were lower than our optimal yield^37^. In addition, the amounts of P fertilizer or K fertilizer applied in previous studies were greater than those applied in our study, with P > N > K or K > P > N^38,39^. These results demonstrate that regions with different soil environments or climates and varieties with different characteristics necessitate different fertilizer combinations. It was evident that the present study was conducted under relatively semi-arid and low-fertility conditions, and the results were obtained with early-maturing varieties. If rain is abundant, fertility is high, and mid–late-maturing varieties are used, the amount of P and K fertilizers applied should be appropriately increased, and the amount of N fertilizer should be reduced. Therefore, the yield response of different adzuki bean varieties in different regions to NPK fertilizer needs to be further explored.

In our production trials, the optimal fertilizer combination achieved results predicted by the model in Baicheng, although the same combination in the other three locations (Tongyu, Taonan and Zhenlai) did not, as these sites were affected by sandy loam soils and low-moisture conditions; however, the yields were still significantly greater than those in response to normal fertilizer. Our optimal fertilizer combination resulted in increased yields at multiple production sites in the region and could serve as a reference for producing high-yield-inducing fertilizer in semi-arid areas of other regions. Optimal fertilizer models for high production in normal regions could be investigated on the basis of this reference.

An economical fertilizer combination was established on the basis of the best fertilizer combination. In consideration of the market fluctuation with respect to the cost of fertilizer and seed, a fertilizer model inclusive of cost-effectiveness was constructed to obtain high yields and economic benefits. The corresponding amounts of fertilizer were 57.6 kg ha^−1^ of N, 47.03 kg ha^−1^ of P_2_O_5_, and 31.64 kg ha^−1^ of K_2_O, although less K fertilizer should be applied than the optimal amount because of the relatively high price of K fertilizer. This economical fertilizer combination could promote the use of profitable fertilizer in future production of adzuki bean.

## Conclusions

In our field research, the effects of various combinations of N, P, and K on yield and yield components were significant. According to the fertilizer model, K fertilization is an important strategy in the production of adzuki bean, as the yield and yield components were more sensitive to K fertilizer than to N and P fertilizers. The high yield achievements were intense at the 0.5 to 1.34 levels under the single and interaction effects and under the effects of the optimal combination of N, P, and K fertilizers. The effects of the combinations of N, P, and K fertilizers significantly influenced the yield more than the single or interaction effects of N, P, and K fertilizers did. The optimal fertilizer combination for achieving high yield comprised 57.23–68.43 kg ha^−1^ N, 36.04–47.32 kg ha^−1^ P_2_O_5_ and 50.29–61.27 K_2_O. The fertilizer resulting in the maximum yield (N:P_2_O_5_:K_2_O = 1:0.75:0.95) comprised 62.98 kg ha^−1^ N, 47.04 kg ha^−1^ P_2_O_5_, and 59.95 kg ha^−1^ K_2_O; although this was the best combination, it approached the upper limit of the optimal fertilizer combination. The optimal fertilizer combination was successful in terms of production at multiple sites with chernozem and sandy loam soils. Thus, our research could provide a reference for producing high-yield-inducing fertilizer in semi-arid areas. The most economical fertilizer comprises 57.60 kg ha^−1^ N, 47.03 kg/ha^−1^ P_2_O_5_, and 31.64 kg ha^−1^ K_2_O, and its optimum ratio is N:P_2_O_5_:K_2_O = 1:0.82:0.55. The economical fertilizer combination could promote profitable fertilizer in the future production of adzuki bean.

## Materials and Methods

### Geographic design

Five-year-long (2014–2018) trials were conducted around the Kerqin grassland square, and four test sites were established in Baicheng, Zhenlai, Taonan and Tongyu of Jilin Province, China (one site at each location). The territory is the main production region of China. The test region had two soil types with low soil fertility. The soil at Baicheng was a chernozem, and the other three locations had sandy loam soils. This area has a temperate semi-arid continental monsoon climate, and the longitude and latitude of the area are 123° E, 45° N, respectively. The region has a daily mean temperature of 20 °C (0.8 °C above the average for the area), an annual sunshine duration of 1243.2 h, and an annual mean rainfall of 404.9 mm.

### Field research data collection

Field trials were performed from 2014–2016 using the early-maturing adzuki bean variety Baihong 4 at the Baicheng Academy of Agricultural Sciences, Baicheng (45.62° N; 122.81° E), Jilin Province, China. The field trial consisted of 23 treatments of various N, P, and K combinations using a three-factor and five-level quadratic orthogonal rotation combination design (Table 4); this setup was in accordance with that of our previous study^40^. All treatments were repeated three times in completely randomized blocks for a total of 69 test plots. Each plot was 12 square metres in area and contained four rows, with ten seedlings metre^−1^ at a line spacing of 15 cm, and the row spacing was 60 cm. At the two-leaf stage, the plants were thinned to a uniform density of 180,000 plants ha^−1^. The fertilizers were mixed and sprayed as seed fertilizers to a depth of 5 cm before the seeds were sown during middle May. The N fertilizer (urea containing 46% N and diammonium phosphate containing 17% N), P fertilizer (calcium superphosphate containing 12% P_2_O_5_ and diammonium phosphate containing 47% P_2_O_5_), and K fertilizer (potassium sulphate containing 50% K_2_O) used were obtained from Sinochem Jilin Changshan Fertilizer Co., Ltd. (Song Yuan, Jilin Province, China).

**Table 4:** N, P, and K factor levels for adzuki bean. X_1j_, X_2j_, and X_3j_ correspond to levels of N, P_2_O_5_ and K_2_O.

Factors	Change interval (kg ha^−1^)	Experimental Levels (kg ha^−1^)					Code formula
		+1.68	+1	0	−1	−1.68	
N (X_1_)	23.48	78.99	62.98	39.50	16.02	0.00	X_1_ = (X_1j_*23.48) + 39.50
P_2_O_5_ (X_2_)	17.54	59.00	47.04	29.50	11.96	0.00	X_2_ = (X_2j_*17.54) + 29.50
K_2_O (X_3_)	22.35	75.19	59.95	37.60	15.25	0.00	X_3_ = (X_3j_*22.35) + 37.60

**Figure 6:**
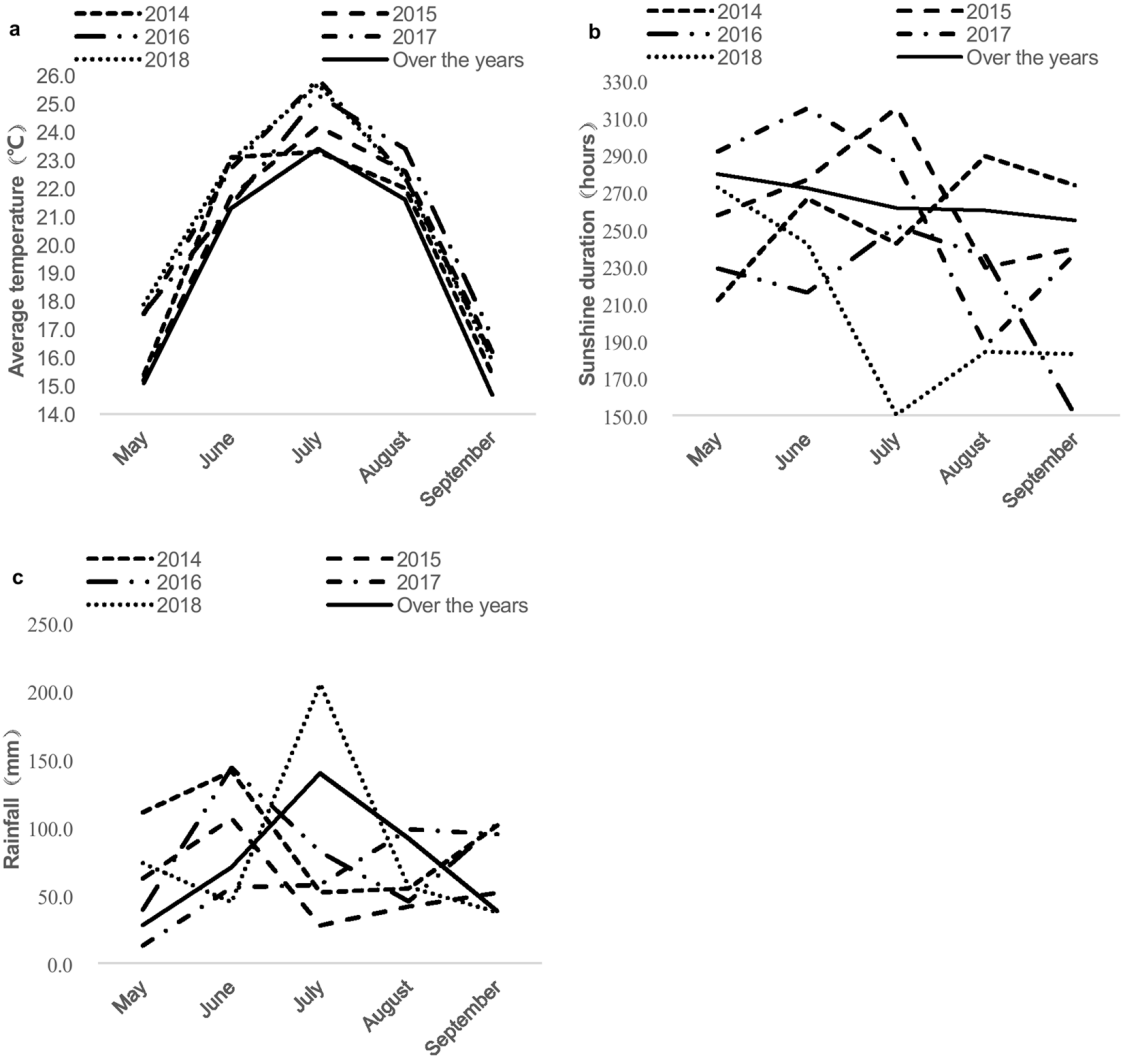
Meteorological data of Baicheng city, jilin province, China, from 2014 to 2018. (**a**) Average temperature (**b**) Sunshine duration, (**c**) Rainfall.

At the growth periods of the trails in 2014–2016, the temperature and sunshine duration were not affected yield achievement, with the proper field management (Fig. 6a, b). During the flowering stage of July and August in 2014, 2015 and 2016, there was less rainfall than there had been in the corresponding months of previous years. Consequently, irrigation was applied once in July and then again in August (Fig. 6c). Samples were hand-harvested when the plants reached maturity from each field plot. The yield and yield components were determined according to the metrics described in our previous work^40^.

### Model description

On the basis of the yield and yield components in response to the 23 treatments of N, P, and K fertilizers in the field research (Table 1), regression equations were constructed to determine the relationships between each of N, P, and K fertilizers and the yield and yield components. Variance analyses were performed to test the fits of the equation models and to test the significance of the effects of the N, P, and K fertilizers on the yield and yield components. The single and interaction effects of the N, P, and K fertilizers were explored in addition to the three-factor combinations. The variable frequency distributions projected the performances of N, P, and K fertilizers in optimal fertilizer combinations in terms of high yield and yield components, and the 95% confidence intervals were calculated for the optimal N, P, and K fertilizer intervals.

### Model implementation

The production trials were conducted during 2017–2018 in Baicheng, Zhenlai, Taonan, and Tongyu, Jilin Province, China. The optimal fertilizer combination for maximum yield (N:P_2_O_5:_K_2_O = 1:0.75:0.95) was applied in the trails. This fertilizer comprised optimized components of 100 kg ha^−1^ of urea, 100 kg ha^−1^ of diammonium phosphate, and 120 kg ha^−1^ of potassium sulphate. Normal compound fertilizer (N:P_2_O_5_:K_2_O = 1:1:1) was applied at a rate of 300 kg ha^−1^ for comparison. The production trial areas were 0.5 ha in each location. The Zhenlai, Taonan, and Tongyu are affiliated to Baicheng, and the climate conditions are similar. At the growth periods of the trails in 2017–2018, the temperature and sunshine duration were not affected yield production, with the proper management (Fig. 6a,b). During the flowering stage of July 2017 and August 2018, there was less rainfall than there had been in previous years. Consequently, irrigation was applied once in July 2017 and in August 2018 (Fig. 6c).

### Data analysis

Microsoft Excel 2010 was used for data processing and table and figure construction. Data Processing System (DPS) software (Hangzhou Ruifeng Information Technology Co., Ltd., Hangzhou, China) was used for designing the quadratic orthogonal rotation combination and for performing the variance analyses and regression analyses.
